# DNA/RNA helicase DHX36 is required for late stages of spermatogenesis

**DOI:** 10.1093/jmcb/mjac069

**Published:** 2022-12-09

**Authors:** Kejia Zhang, Tianxin Zhang, Yujie Zhang, Jinyu Yuan, Xinzhe Tang, Chaobao Zhang, Qianqian Yin, Yonglian Zhang, Ming-Han Tong

**Affiliations:** State Key Laboratory of Molecular Biology, Shanghai Key Laboratory of Molecular Andrology, Shanghai Institute of Biochemistry and Cell Biology, Center for Excellence in Molecular Cell Science, University of Chinese Academy of Sciences, Chinese Academy of Sciences, Shanghai 200031, China; State Key Laboratory of Molecular Biology, Shanghai Key Laboratory of Molecular Andrology, Shanghai Institute of Biochemistry and Cell Biology, Center for Excellence in Molecular Cell Science, University of Chinese Academy of Sciences, Chinese Academy of Sciences, Shanghai 200031, China; State Key Laboratory of Molecular Biology, Shanghai Key Laboratory of Molecular Andrology, Shanghai Institute of Biochemistry and Cell Biology, Center for Excellence in Molecular Cell Science, University of Chinese Academy of Sciences, Chinese Academy of Sciences, Shanghai 200031, China; State Key Laboratory of Molecular Biology, Shanghai Key Laboratory of Molecular Andrology, Shanghai Institute of Biochemistry and Cell Biology, Center for Excellence in Molecular Cell Science, University of Chinese Academy of Sciences, Chinese Academy of Sciences, Shanghai 200031, China; State Key Laboratory of Molecular Biology, Shanghai Key Laboratory of Molecular Andrology, Shanghai Institute of Biochemistry and Cell Biology, Center for Excellence in Molecular Cell Science, University of Chinese Academy of Sciences, Chinese Academy of Sciences, Shanghai 200031, China; State Key Laboratory of Molecular Biology, Shanghai Key Laboratory of Molecular Andrology, Shanghai Institute of Biochemistry and Cell Biology, Center for Excellence in Molecular Cell Science, University of Chinese Academy of Sciences, Chinese Academy of Sciences, Shanghai 200031, China; State Key Laboratory of Molecular Biology, Shanghai Key Laboratory of Molecular Andrology, Shanghai Institute of Biochemistry and Cell Biology, Center for Excellence in Molecular Cell Science, University of Chinese Academy of Sciences, Chinese Academy of Sciences, Shanghai 200031, China; State Key Laboratory of Molecular Biology, Shanghai Key Laboratory of Molecular Andrology, Shanghai Institute of Biochemistry and Cell Biology, Center for Excellence in Molecular Cell Science, University of Chinese Academy of Sciences, Chinese Academy of Sciences, Shanghai 200031, China; School of Life Science, Hangzhou Institute for Advanced Study, University of Chinese Academy of Sciences, Chinese Academy of Sciences, Hangzhou 310024, China; State Key Laboratory of Molecular Biology, Shanghai Key Laboratory of Molecular Andrology, Shanghai Institute of Biochemistry and Cell Biology, Center for Excellence in Molecular Cell Science, University of Chinese Academy of Sciences, Chinese Academy of Sciences, Shanghai 200031, China

**Keywords:** *Dhx36*, meiosis, guanine-quadruplex (G4), *Spo11*, synapsis, meiotic recombination, crossover

## Abstract

Spermatogenesis is a highly complex developmental process that typically consists of mitosis, meiosis, and spermiogenesis. DNA/RNA helicase DHX36, a unique guanine-quadruplex (G4) resolvase, plays crucial roles in a variety of biological processes. We previously showed that DHX36 is highly expressed in male germ cells with the highest level in zygotene spermatocytes. Here, we deleted *Dhx36* in advanced germ cells with *Stra8*-GFPCre and found that a *Dhx36* deficiency in the differentiated spermatogonia leads to meiotic defects and abnormal spermiogenesis. These defects in late stages of spermatogenesis arise from dysregulated transcription of G4-harboring genes, which are required for meiosis. Thus, this study reveals that *Dhx36* plays crucial roles in late stages of spermatogenesis.

## Introduction

Spermatogenesis in mammals, such as the lab mouse, is a highly specialized and coordinated differentiation process that produces mature haploid spermatozoa (Nishimura and L'Hernault, 2017). It typically consists of three successive phases: mitosis, meiosis, and spermiogenesis (Nishimura and L'Hernault, 2017). During the mitotic phase, spermatogonial stem cells either undergo self-renewal to maintain stem cell pool or differentiate to ultimately form type B spermatogonia through successive cell divisions. Type B spermatogonia then give rise to preleptotene spermatocytes that enter meiotic phase. In meiosis, spermatocytes undergo one round of replication followed by two consecutive rounds of chromosome segregation, meiosis I and meiosis II. During meiotic prophase I, chromosomes undergo homolog recombination, pairing, and synapsis. Recombination is initiated by programmed DNA double-strand break (DSB) formation at hotspots (Keeney and Neale, 2006; de Massy, 2013; Pratto et al., 2014; Robert et al., 2016; Vrielynck et al., 2016; Bhattacharyya et al., 2019). Meiotic DSBs are generated by the topoisomerase II-like SPO11 and GM960 proteins together with several protein partners, including IHO1, MEI4, and REC114 (Romanienko and Camerini-Otero, 2000; Robert et al., 2016; Vrielynck et al., 2016; Bhattacharyya et al., 2019). These DSBs must subsequently repaired by the replication protein A (RPA), DMC1, and Rad51 with a number of cofactors to generate either crossovers (COs) or non-COs (Ribeiro et al., 2016; Hinch et al., 2020). While homologs separate at meiosis I, sister chromatids segregate to give rise to haploid round spermatids during meiosis II (Chambon et al., 2013; Wassmann, 2013). Resulting round spermatids further undergo extensive morphologic and molecular changes to develop into elongated mature spermatozoa during spermiogenesis (Poccia, 1986; Shi et al., 2013). These processes are accurately controlled by gene expression programs at transcriptional, posttranscriptional, and translational levels (Lin and Tong, 2019).

Guanine-quadruplex (G4) is a four-stranded tetrad structure that is spontaneously generated by guanine-rich nucleic acid sequences, which have been found highly abundant in both DNA and RNA (Bannister et al., 2004; Paeschke et al., 2011; Henderson et al., 2014; Varshney et al., 2020; Wu et al., 2021). Once formed, G4 structures are highly stable and are likely to impede DNA and RNA metabolism (Bochman et al., 2012; Paeschke et al., 2013; Fay et al., 2017). Experiments that can directly detect G4 structures have identified that they are prevalent in genome and in RNA *in vivo* (Biffi et al., 2013, 2014; Henderson et al., 2014; Moye et al., 2015). Accumulating evidences reveal that G4 structures play critical roles in numerous cellular processes, including telomere maintenance, transcription, splicing, translation, DNA replication, DNA damage, and genome stability (Sen and Gilbert, 1988; Creacy et al., 2008; Booy et al., 2012; Moye et al., 2015; Rhodes and Lipps, 2015; Weldon et al., 2017; Varshney et al., 2021). As might be expected, both formation and resolution of G4 structures require to be spatiotemporally controlled in a cellular context (Mendoza et al., 2016; Tang et al., 2016; Tippana et al., 2016; Ketkar et al., 2017; Sauer and Paeschke, 2017; van Wietmarschen et al., 2018). For example, helicases are able to resolve G4 structures, whereas several specific protein chaperons can promote G4 formation. DHX36 (also named as RHAU or G4R1), a member of the DEAH/RHA family of DNA and RNA helicase, is capable of binding and unfolding both DNA and RNA G4 structures with high affinity (Gueddouda et al., 2017). Recent studies suggested that G4 binding induces rearrangements of the DHX36 helicase core, which pulls the single-stranded DNA (ssDNA) tail nucleotide residues one by one, thereby unwinding the DNA G4 (You et al., 2017; Chen et al., 2018a). It has also been shown that DHX36 can unwind RNA G4 structures formed at the 5′UTR to promote translation (Bugaut and Balasubramanian, 2012; Chen et al., 2021). *Dhx36* is an evolutionary conserved gene that has been shown ubiquitous expression in human and mouse tissue (Lai et al., 2012). These results suggest a crucial role of *Dhx36* in G4-mediated functions and development. Accordingly, genetic deletion studies showed that *Dhx36* was required for early embryogenesis, cardiac development, skeletal muscle regeneration, and hematogenesis (Booy et al., 2012; Lai et al., 2012; Nie et al., 2015; Chen et al., 2021).

Deletion of *Dhx36* in gonocytes at embryonic day 15.5 using Vasa-Cre resulted in blockage of spermatogonial differentiation (Gao et al., 2015). Because male germ cells are depleted before meiosis in this model, it remains unknown whether *Dhx36* functions in late stages of spermatogenesis. To address this question, we here deleted *Dhx36* in type A1 spermatogonia using a *Stra8*-GFPCre knock-in mouse line (Lin et al., 2017). We demonstrated that germ cell-specific disruption of *Dhx36* causes meiotic defects and impaired spermiogenesis, leading to male infertility. We further show that *Dhx36*-deficient spermatocytes failed to complete meiosis because of deregulation of crucial regulators implicated in meiotic recombination. Moreover, we indicate that DHX36 could regulate gene expression through unfolding G4 structures in the promoter to potentially govern male germ cell development. This work thus reveals a crucial role of *Dhx36* in G4-mediated transcription during the late stages of spermatogenesis.

## Results

### Germ cell-specific inactivation of Dhx36 causes male sterility from impaired spermatogenesis

We previously showed that *Dhx36* mRNA was dynamically expressed in male germ cells and reached its peak in zygotene spermatocytes (Supplementary Figure S1A; Chen et al., 2018b). Consistent with the mRNA expression, we found that DHX36 protein was predominantly detected in primary spermatocytes by immunofluorescence staining (Supplementary Figure S1B), suggesting a role for DHX36 in spermatogenesis, in particular meiosis. It has recently been demonstrated that *Dhx36* could play a role in spermatogonial differentiation (Gao et al., 2015). To determine whether *Dhx36* functions on late stages of spermatogenesis such as meiosis and spermiogenesis, a germ line-specific knockout mouse model (*Dhx36*^fl/del^,*Stra8*-GFPCre; designated as *Dhx36-*cKO) was used by crossing a conditional mouse in which exon 8 of the *Dhx36* allele are flanked by loxP sites (*Dhx36*^fl/fl^) with a *Stra8*-GFPCre mouse to specifically delete *Dhx36* alleles from type A1 spermatogonia onward (Supplementary Figure S1C; Lin et al., 2017). We used the two different genotypes of mice as controls: *Dhx36*^fl/del^ and *Dhx36*^fl/+^,*Stra8*-GFPCre.

**Figure 1:**
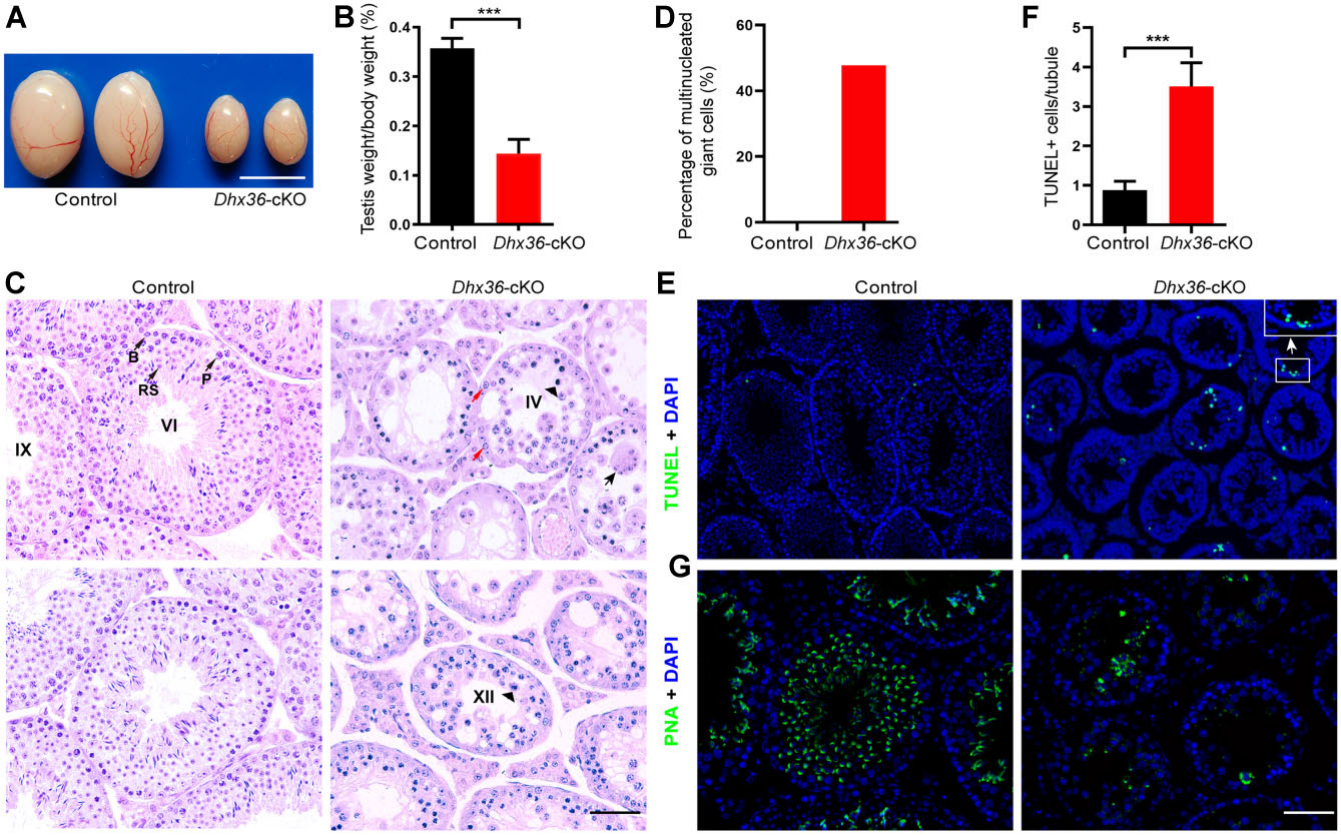
Germ cell-specific *Dhx36* deficiency leads to impaired spermatogenesis. (**A**) Gross morphology of representative testes from 8-week-old control and age-matched *Dhx36*-cKO mice. Scale bar, 2 mm. (**B**) Comparison of testicular weight from controls and *Dhx36-*cKO mutants (*n* = 6 for each genotype). Data are expressed as mean ± SD. ***P < 0.001 by Student's *t*-test. (**C**) H&E staining of control (left) and *Dhx36-*cKO (right) testes at 8 weeks of age. The upper left panel shows an epithelial stage VI tubule in a control testis section, which contains several layers of spermatogenic cells at different developmental stages: B, type B spermatogonia; P, mid-pachytene spermatocytes; RS, round spermatids; and elongating spermatids. In the right panels, arrowheads indicate apoptotic spermatocytes at epithelial stage IV (upper), which was identified by the presence of late intermediate spermatogonia (red arrows), and at epithelial stage XII (lower) in *Dhx36* mutants. The black arrow indicates large multinucleated giant cells in *Dhx36* mutants (upper right panel). Scale bar, 20 μm. (**D**) Comparison of multinucleated giant cells in seminiferous tubules from controls and mutant testes at 8 weeks of age (*n* = 3 for each genotype). (**E**) Detection of apoptotic spermatogenic cells (TUNEL assay, green signal) from control (left) and age-matched *Dhx36-*cKO mutant testes, with co-staining for DAPI. Scale bar, 20 μm. (**F**) Quantification of TUNEL-positive cells per seminiferous tubule in control and mutant testes at 8 weeks of age (*n* = 3 for each genotype). Data are expressed as mean ± SD. ***P < 0.001 by Student's *t*-test. (**G**) Fluorescence staining of testis sections from adult control and *Dhx36-*cKO mice with fluorescence dye-labelled peanut lectin (PNA, green) for acrosomes and DAPI (blue). Scale bar, 20 μm.

Although *Dhx36-*cKO male mice exhibited normal copulating behavior, they were completely sterile. Testes from *Dhx36*-cKO mice were much smaller than those of the littermate controls (Figure 1A and B). Hematoxylin and eosin (H&E) staining from adult control testes showed the full array of spermatogenic cells, including spermatogonia, spermatocytes, and round and elongated spermatids (Figure 1C). In contrast, we found that *Dhx36-*cKO seminiferous tubules were largely if not completely devoid of postmeiotic cells, but nearly half (∼47.8%) of tubules contained many multinucleated giant cells (Figure 1D). Close examination of the mutant testes exhibited that some spermatocytes underwent apoptosis at stages IV equivalent to mid-pachytene (Ahmed and de Rooij, 2009), whereas many spermatocytes could progress to the first meiotic metaphase, where they could arrest at stage XII, as most metaphase spermatocytes underwent apoptosis, indicating severe meiotic defects (Figure 1C). Terminal deoxynucleotidyl transferase-mediated dUTP nick-end labelling (TUNEL) staining further confirmed more frequent apoptotic spermatocytes in mutants (Figure 1E and F). Staining of FITC-conjugated PNA, capable of binding the acrosomal membrane, displayed that multinucleated giant cells in mutants are mainly composed of abnormal step 1–step 3 round spermatids, revealing a complete arrest of spermiogenesis before step 3 spermatids (Figure 1G). Consistent with this, no sperm was found in the epididymis of adult mutants (Supplementary Figure S1D). Altogether, these results demonstrated that *Dhx36* plays an important role in late stages of spermatogenesis, and its inactivation results in meiotic and spermiogenesis defects. Thus, we concluded that *Dhx36-*cKO male sterility could result from impaired spermatogenesis.

### Dhx36 deficiency leads to synapsis failures

**Figure 2:**
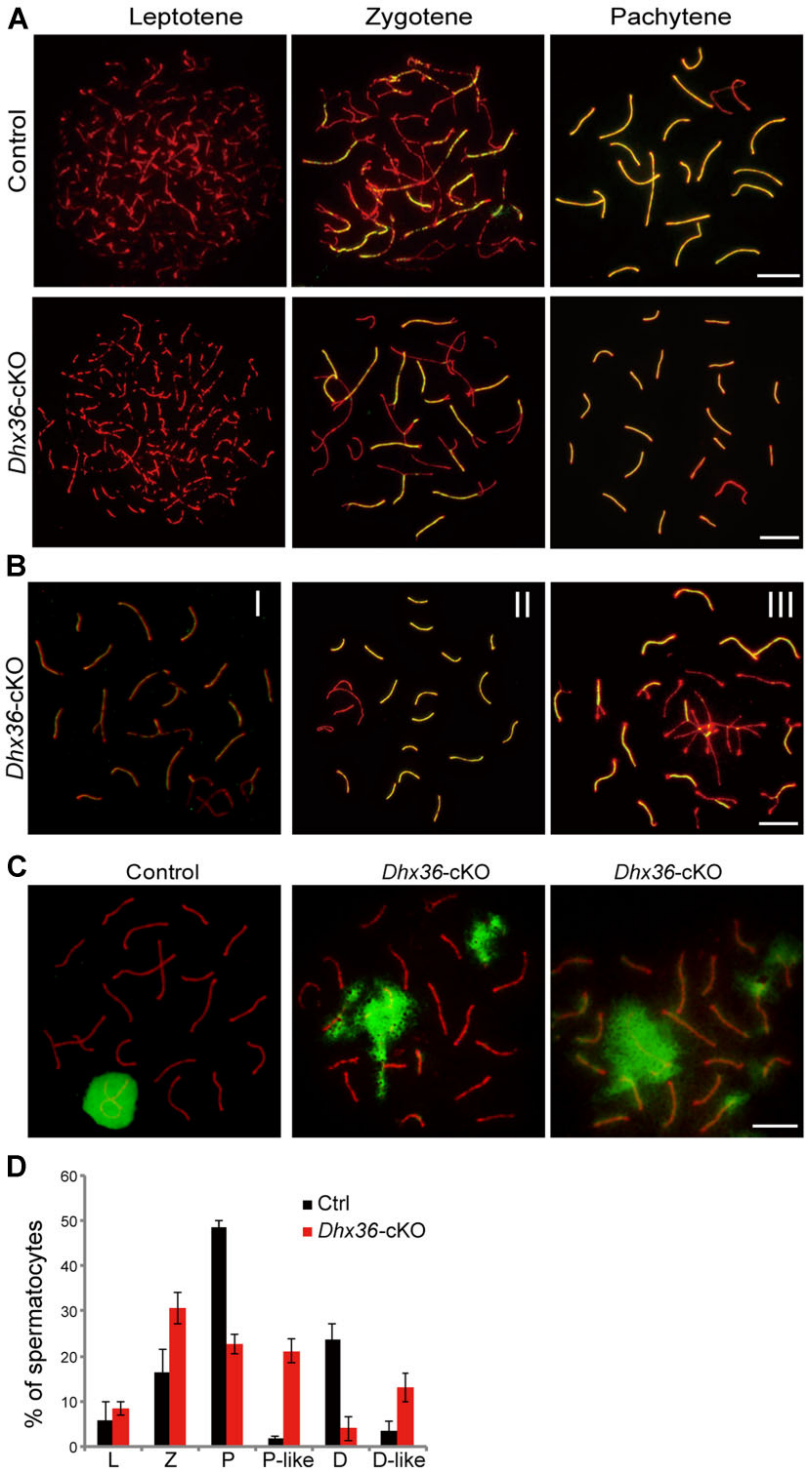
Synapsis is defective in *Dhx36-*cKO mutant spermatocytes. (**A**) Chromosome spreads of control and *Dhx36-*cKO mutant spermatocyte nuclei immunostained for SYCP1 (green) and SYCP3 (red). (**B**) Representative aberrant synaptic spermatocytes from *Dhx36-*cKO mutant testes immunostained for SYCP1 (green) and SYCP3 (red). (**C**) Spread nuclei of control and *Dhx36-*cKO mutant spermatocyte immunostained for γH2AX (green) and SYCP3 (red). (**D**) Quantification of the frequencies of meiotic spermatocyte in control and *Dhx36-*cKO mutant testes at 8 weeks of age (*n* = 3 for each genotype). L, leptotene; Z, zygotene; P, pachytene; P-like, pachytene-like; D, diplotene, D-like, diplotene-like. Scale bars, 10 μm.

*Dhx36* could contribute to multiple processes during spermatogenesis as a mix of phenotypes in mutants, i.e. incomplete penetrance of pachytene arrest, metaphase I arrest, and complete arrest in round spermatids as described earlier. To explore the function of *Dhx36* in meiosis, we first performed spermatocyte nuclear spreading with immunostaining for SYCP3, the axial/lateral element protein, and SYCP1, the central element protein of synaptonemal complex (SC) (Hamer et al., 2006, 2008). Synapsis is one of the hallmarks of meiotic prophase I progression (Zickler and Kleckner, 2015). The development of chromosome axes and the SC can be used to define the substages of meiotic prophase I (Zickler and Kleckner, 2015). Discrete dots of SYCP3 signal initially distribute to chromosomal axes at the leptotene spermatocytes, and progressively form continuous filaments along the aligning axes at the zygotene (Hamer et al., 2006, 2008). In pachytene spermatocytes, SYCP1 assembled to the full length between all autosome homologs and the paired region between the pseudoautosomal region of the X and Y chromosomes (de Vries et al., 2005; Kauppi et al., 2011). We found that chromosome axis development and SC formation of leptotene and zygotene spermatocytes were comparable in *Dhx36-*cKO and control mice. Although complete synapsis was observed in most of mutant spermatocytes (Figure 2A), some spermatocytes displayed aberrant synaptic features and arrested at a stage termed ‘pachytene-like’ equivalent to late zygotene or early pachytene spermatocytes in controls (Figure 2B). Aberrant nuclei displayed either (i) incomplete synapsed homologs (Figure 2B, I), (ii) a mix of fully synapsed homologs plus asynaptic homolog(s) (Figure 2B, II), or (iii) ‘chromosome tangles’ consisting of synapsed non-homolog(s), asynaptic homolog(s), and fully synapsed homologs (Figure 2B, III). Such defective synapsis provides a plausible explanation for increased apoptotic spermatocytes in mutants.

We then stained spermatocyte nuclear spreads with antibodies against SCP3 and phosphorylated H2AX at Ser139 (γH2AX), a marker for DSBs. γH2AX signals occurs from leptotene spermatocytes because of DSB formation, then decrease during zygotene as DSBs are repaired (Zickler and Kleckner, 2015). As meiosis proceeds to the pachytene and diplotene stages, γH2AX is only localized to the sex body and disappears from synapsed autosomes (Turner, 2007). We found that leptotene and zygotene spermatocytes from both mutants and controls showed similar γH2AX signals and dynamics. However, we observed that pachytene-like spermatocytes in *Dhx36-*cKO mice exhibited high levels of persistent γH2AX signals at asynaptic regions, which are similar to γH2AX staining at the sex body in control pachytene (Figure 2C). These results indicate a failure in the repair of mutant meiotic DSBs. Such defective DSB repair could cause apoptosis in mutant spermatocytes.

To monitor the development of spermatocytes in detail, we also counted the number of individual substages of spermatocytes in meiotic prophase I from adult mutant and control mice (Figure 2D). We observed a significant increase in the proportion of zygotene spermatocytes (16.40% ± 5.30% vs. 30.73% ± 3.47%; mean ± SD; *P* < 0.01, two-tailed *t-*test) as well as marked decreases in pachytene (48.47% ± 1.72% vs. 22.63% ± 2.20%; mean ± SD; *P* < 0.001, two-tailed *t-*test) and diplotene (23.83% ± 3.43% vs. 4.03% ± 2.65%; mean ± SD; *P* < 0.001, two-tailed *t-*test) spermatocytes in mutants relative to controls (Figure 2D). In addition, we found significant increases in the percentage of pachytene-like (1.97% ± 0.45% vs. 21.20% ± 2.50%; mean ± SD; *P* < 0.01, two-tailed *t-*test) and diplotene-like (3.47% ± 2.15% vs. 12.97% ± 3.15%; mean ± SD; *P* < 0.01, two-tailed *t-*test) spermatocytes in mutant mice compared to control mice.

Taken together, these results reveal that inactivation of *Dhx36* leads to synaptic defects and unrepaired DSBs, which result in apoptosis of pachytene spermatocytes during meiotic prophase I.

### Dhx36 deficiency results in dysregulated recombination

**Figure 3:**
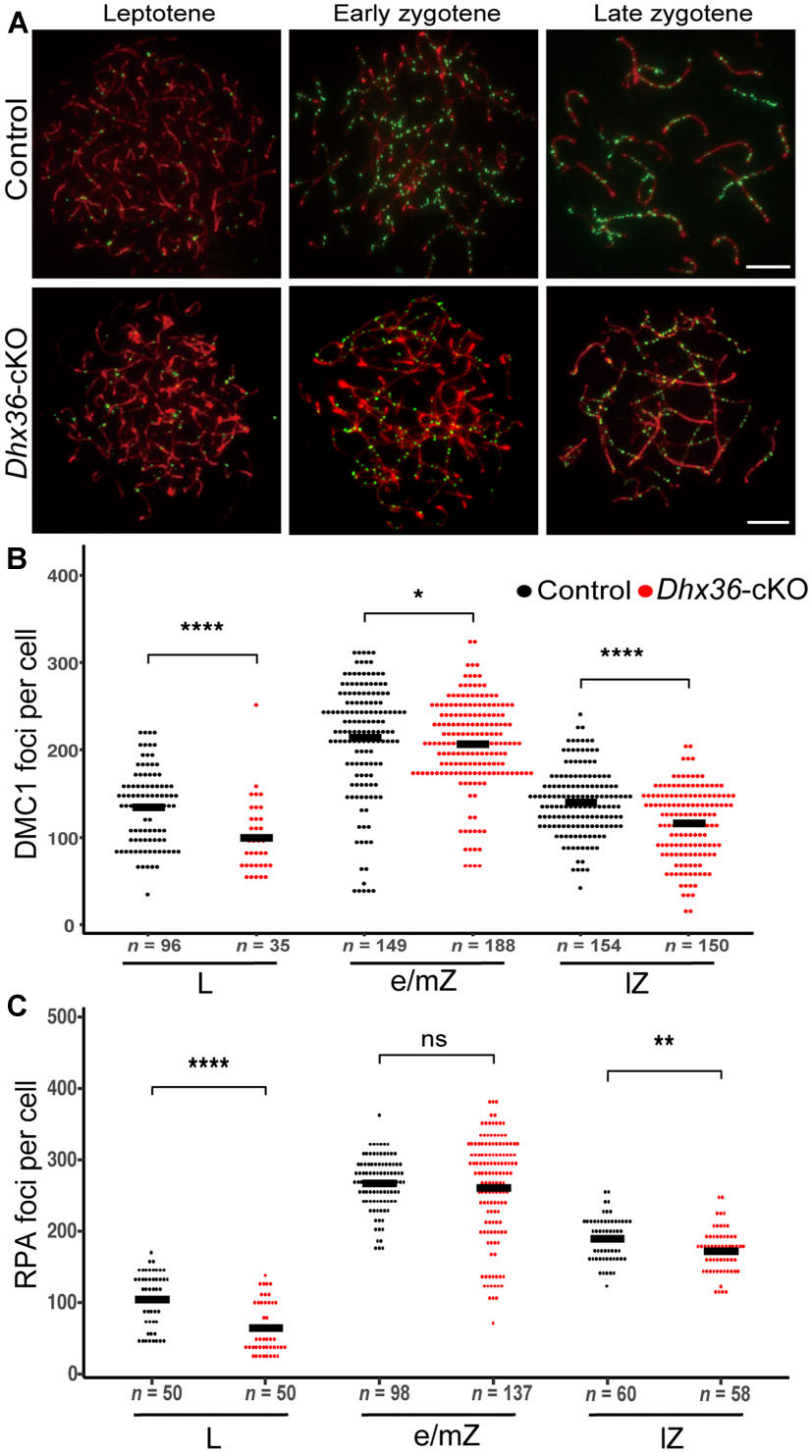
*Dhx36* deficiency results in dysregulated recombination. (**A**) Representative images of immunostaining of DMC1 (green) and SYCP3 (red) in spread nuclei of control and *Dhx36-*cKO mutant spermatocytes. Scale bar, 10 μm. (**B** and **C**) Quantification of DMC1 and RPA focus numbers in spermatocyte at leptotene (L), early/middle zygotene (e/mZ), and late zygotene (lZ) from control and *Dhx36-*cKO mutant testes (*n* = 3 for each genotype). Each dot in the graphs represents the number of DMC1 (**B**) or RPA (**C**) foci per nucleus. Data are expressed as mean ± SD. *P < 0.05, **P < 0.01, ****P < 0.0001; ns, no significant difference. Student's *t*-test, *n* = number of cells analyzed.

DSBs are generated by SPO11 and GM960 and are then resected to form 3′ ssDNA ends that are bound by RPA and the recombinases RAD51 and DMC1 to mediate stand invasion into the homologous chromosome (Romanienko and Camerini-Otero, 2000; Ribeiro et al., 2016; Robert et al., 2016; Vrielynck et al., 2016; Hinch et al., 2020). As meiotic recombination progresses, the levels of RPA, RAD51, and DMC1 on DSB repair intermediates dynamically change (Hinch et al., 2020). DMC1 and RAD51 foci first occur at leptonema, reach the peak at early/mid-zygonema, and then decline substantially by late zygonema (Hinch et al., 2020). Thus, DMC1 and RPA foci could represent early meiotic recombination events. To assess whether meiotic recombination is affected in *Dhx36-*cKO male mice, we first determine the numbers of DMC1 foci on meiotic chromosome axis in *Dhx36-*cKO and control mouse spermatocytes. The numbers of DMC1 foci were significantly decreased in mutant leptotene spermatocytes compared with controls. Fewer foci were also present at both early/middle and late zygonema in mutant spermatocytes (Figure 3A). However, we observed DMC1 foci accumulated to asynaptic axes at pachytene-like spermatocytes in mutants, while the number of DMC1 foci decreased at synapsed chromosomes as in controls (Figure 3B). The ssDNA-binding protein RPA foci showed similar pattern to DMC1 foci in mutant spermatocytes (Figure 3C). These results imply that DSB generation and/or resection could be reduced or delayed by *Ddx36* deficiency in male germ cells.

### Dhx36-deficient spermatocytes generates fewer COs

**Figure 4:**
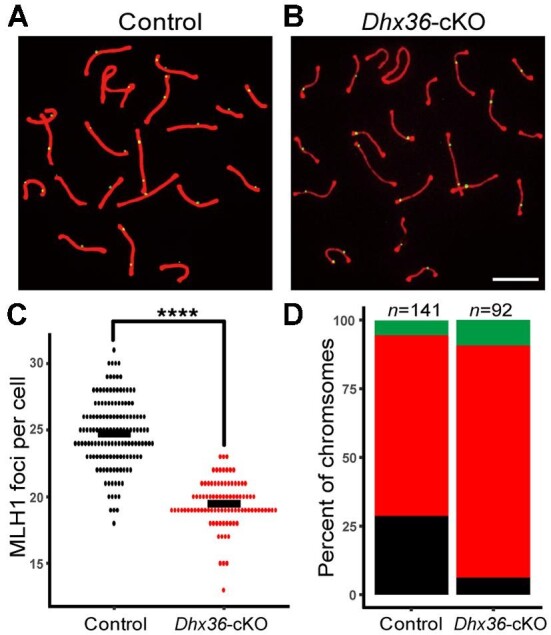
*Dhx36-*deficient mice display CO defects. (**A** and **B**) Re-presentative images of nuclear spreads of mid-pachytene to late pachytene spermatocytes from control (**A**) and *Dhx36-*cKO mutant (**B**) testes stained with anti-SYCP3 (Red) and anti-MLH1 (green) antibodies. Scale bar, 10 μm. (**C**) Quantification of MLH1 focus numbers in mid-pachytene to late pachytene spermatocytes in control and *Dhx36-*cKO mutant testes at 8 weeks of age (*n* = 3 for each genotype). Each dot in the graph represents the number of DMC1 foci per nucleus. Data are expressed as mean ± SD. ****P < 0.0001 by Student's *t*-test. (**D**) Percent of chromosomes carrying 0 (green), 1 (red), and 2–3 (black) MLH1 foci per homolog in control and *Dhx36-*cKO mutant pachytene spermatocytes.

As described earlier, we observed the mix of stages IV and XII apoptosis in *Dhx36-*cKO testes. CO defects could cause apoptosis of spermatocytes during meiotic metaphase I at stage XII because accurate segregation of homologs depends on COs (Eaker et al., 2002). The formation of at least one CO per homolog ensures their precise segregation (Hughes and Hawley, 2020). MLH1 is thought to make CO sites, and is essential for the formation of most COs at the late stages of recombination (Peterson et al., 2020). To determine whether defective CO formation might account for apoptosis of mutant spermatocytes during metaphase I, we examined the presence of MLH1 foci in mutant and control spermatocytes. We found that the numbers of MLH1 foci at pachynema in mutant mice were significantly lower than that in control mice (Figure 4A–C). In addition, at least one pair of homologs in ∼30% of mutant pachytene spermatocytes showed no MLH1 foci (Figure 4B and D). Such CO failures could cause apoptosis of spermatocytes during metaphase I in *Dhx36-*cKO testes.

### Dhx36 deficiency results in misregulation of crucial regulators during meiosis

**Figure 5:**
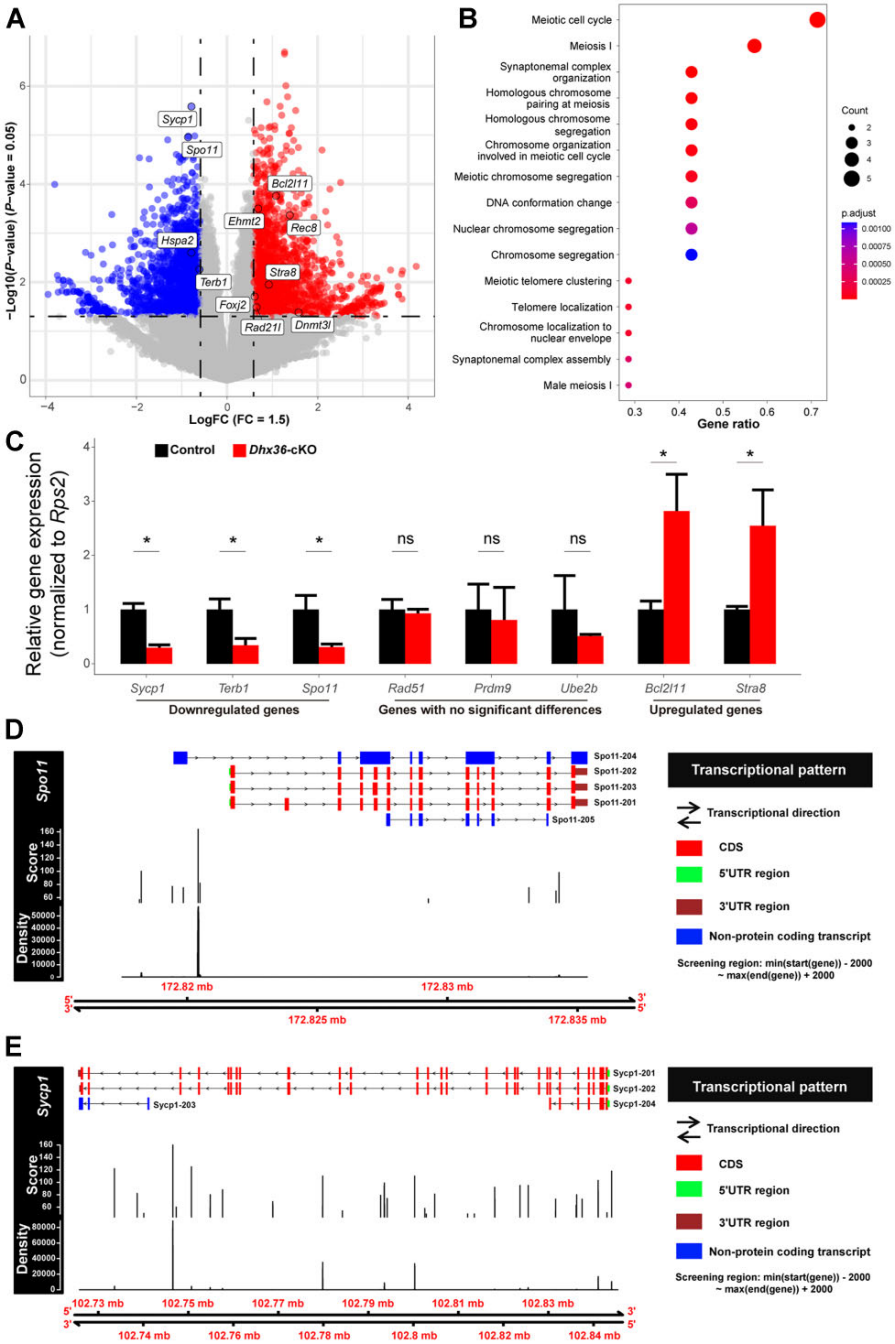
*Dhx36* deficiency leads to alterations of the transcriptome in zygotene spermatocytes. (**A**) Volcano plot showing fold changes of the mRNA levels in zygotene spermatocytes upon *Dhx36* deficiency. (**B**) GO analysis of the DEGs between control and *Dhx36*-cKO mutant zygotene spermatocytes. (**C**) qRT–PCR analysis of the mRNA levels of several genes, including *Sycp1* and *Spo11*, in zygotene spermatocytes of control and *Dhx36*-cKO mutant mice. Data are expressed as mean ± SD from at least two biological replicates. *P < 0.05; ns, no significant difference. (**D** and **E**) The pqsfinder was used to identify potential G4. The diagrams demonstrating PQS score density distribution can help to assess the singularity of PQS distributing along genomic template strand of *Spo11* (**D**) and *Sycp1* (**E**). Promoter region, 2 kb ahead of transcriptional start site, was also included.

To elucidate molecular mechanisms underlying meiotic defects caused by loss of *Dhx36*, we performed high-throughput RNA-sequencing (RNA-seq) to analyze the transcriptome of purified zygotene spermatocytes from control and *Dhx36*-mutant testes. We identified that a total of 3555 differentially expressed genes (DEGs; 1826 upregulated and 1729 downregulated) exhibited significant changes (*P*-value <0.05, >1.5-fold difference) in mutant zygotene spermatocytes compared to that in controls (Figure 5A; Supplementary Table S1). Gene ontology (GO) analysis of the top-ranked genes indicated an enrichment in genes associated with meiotic cell cycle, homologous chromosome pairing, and chromosome segregation (Figure 5B). It is of note that expression of several crucial regulators for meiotic prophase I, including *Spo11, Sycp1*, and *Terb1*, was markedly reduced upon *Dhx36* deficiency (Figure 5A). The downregulation of *Spo11* and *Sycp1* was further validated by quantitative reverse transcription–polymerase chain reaction (qRT–PCR) on RNA from isolated spermatocytes in controls and mutants (Figure 5C). Consistent with the reduction of DMC1 foci, the decreased expression of *Spo11* could lead to impaired DSB generation in mutants. In addition, gene set enrichment analysis (GSEA) of all DEGs displayed that apoptosis pathway was enriched, which coincided with the increased apoptotic spermatocytes in mutants (Supplementary Figure S2). Taken together, these findings indicate that the defects of meiotic recombination caused by *Dhx36* loss could be due to the misregulation of critical regulators for meiosis.

G4 motifs in promoters and/or coding regions could repress transcription of genes (Varshney et al., 2020). Using immunofluorescence staining of the DNA G4 structure-specific antibody, BG4, as previously described (Biffi et al., 2013), we found that spermatogenic cells exhibited nuclear BG4 staining, which was not seen with DNase I treatment or in the absence of a primary BG4 antibody, suggesting that DNA G4 structures might exist in spermatogenic cells (Supplementary Figure S3). Given that DHX36 is a unique ATP-dependent 3′-5′ DEAH-box helicase, which could bind and unwind DNA G4 structure, we next asked whether there is a correlation between misregulated genes in *Dhx36-*deficient spermatocytes and the genes that contain G4 motif. We used pqsfinder, an R/Bioconductor package that can detect potential quadruplex-forming sequences (PQS) in DNA to identify potential G4 motif in the genomic template strand of the key genes (Hon et al., 2017). We found a significant G4 enrichment in the promoter regions of downregulated meiosis-associated genes compared to upregulated meiosis-associated genes in *Dhx36*-deficient spermatocytes (Supplementary Figure S4A). Of note, the downregulation level negatively correlated with the number of potential DNA G4s (Supplementary Figure S4B). Among these genes, pqsfinder revealed that the promoter and its adjacent regions of *Spo11* enriched several putative G4 motifs (Figure 5D), while *Sycp1* carried a number of potential G4 structures in its coding regions (Figure 5E). Thus, DHX36 could unwind G4 motif in the promoter and coding regions of crucial meiotic regulators (i.e. *Spo11* and *Sycp1*) to promote their expression in spermatocytes; however, loss of *Dhx36* resulted in unresolved G4 motifs accumulated in the gene promoters, thereby downregulating expression of G4 DNA-associated genes.

## Discussion

DHX36 is a unique ATP-dependent G4 resolvase that has been involved in G4-mediated transcriptional and posttranscriptional regulation (Lai et al., 2012; Nie et al., 2015; Chen et al., 2021). We previously demonstrated that *Dhx36* was highly expressed in male germ cells, suggesting a potential role in spermatogenesis (Gao et al., 2015). The role of *Dhx36* in spermatogonial differentiation has been described (Gao et al., 2015). However, whether and how *Dhx36* regulates the late stages of spermatogenesis remains to be unexplored. In this study, we elucidated the biological function of *Dhx36* in late stages of spermatogenesis using a *Dhx36*-deficient mouse model via the *Stra8*-GFPCre-mediated *Dhx36* deletion in advanced germ cells, and demonstrated that *Dhx36* is required for the late stages of spermatogenesis. Mechanistically, we found that *Dhx36* loss caused deregulation of crucial meiotic regulators which carry G4 structures in their promoters. This study thus reveals the crucial function of *Dhx36* and its potential mediated G4 resolution in meiosis and spermiogenesis.

Meiosis is essential for sexual reproduction and occurs only in germ cells (Ohkura, 2015). Recombination is the most prominent feature in meiosis which can increase genetic diversity during inheritance (Baudat et al., 2013). Molecular mechanism underlying meiotic recombination has been extensively studied; however, transcriptional and posttranscriptional regulation of genes involved in these processes, particularly in mammals, remains largely unknown (Baudat et al., 2013; Pratto et al., 2014; Zickler and Kleckner, 2015). Our findings indicate that DHX36 might be a transcriptional regulator of meiosis. We found that loss of *Dhx36* in advanced germ cells caused defects of meiotic recombination, including decreased DSBs, aberrant synapsis, and fewer COs. SPO11 is an essential enzyme for DSB formation, and a lower level of SPO11 leads to a decrease in DSB numbers in mammalian spermatocytes (Romanienko and Camerini-Otero, 2000; Kauppi et al., 2013). SYCP1 is a key element for transverse filaments of SC, and loss of *Sycp1* causes a failure of synapsis formation during meiosis (de Vries et al., 2005). We showed that a number of G4 motifs present in the *Spo11* promoter and the *Sycp1* coding regions. Genomic G4s are stable secondary DNA structures that can be physical obstacles and impede the progression of RNA polymerase II and DNA polymerase, resulting in transcription and replication stalling, respectively (Varshney et al., 2020). A number of studies have shown that G4s in the promoter could affect transcription, while G4 in the gene body could inhibit transcriptional elongation (Varshney et al., 2020). Previous studies have demonstrated that DHX36 can remove the DNA G4 structures to promote gene transcription (Lai et al., 2012; Gao et al., 2015). Accordingly, as shown in this study, *Dhx36*-deficient spermatocytes exhibited altered transcriptomes, in particular decreased expression of both *Spo11* and *Sycp1*, suggesting that G4s in the promoter and gene body play potential roles in the regulation of genes involved in meiosis. Analysis of the DNA bound by DHX36 using ChIP–seq will provide direct insights on how DHX36 controls gene expression during meiosis. On the other hand, it has been shown that DHX36 binds and unwinds RNA G4s to regulate RNA translation (Nie et al., 2015; Chen et al., 2021). We cannot exclude that DHX36 might also modulate expression of genes involved in meiosis at posttranscriptional level. It will be of interest to explore these in the future.

Collectively, this work reveals the crucial role of DHX36 in the regulation of meiosis at transcriptional level.

## Materials and methods

### Mice

The conditional knockout alleles for *Dhx36* (*Dhx36*^fl/fl^) and the *Stra8*-GFPCre knock-in mouse line were generated as described previously (Lin et al., 2017). All animals were maintained on the C57BL/6J genetic background. The germ cell-specific deletion of *Dhx36* was produced by crossing *Dhx36*^fl/fl^ mice with the *Stra8*-GFPCre line. Animal experiments were approved by the Animal Care and Use Committee at Shanghai Institute of Biochemistry and Cell Biology, Center for Excellence in Molecular Cell Science, Chinese Academy of Science, and were performed according to the relevant regulations and guidelines.

### Histology, immunofluorescence, and TUNEL staining

Testes were fixed in Bouin's solution at room temperature (RT) or 4% paraformaldehyde (PFA) at 4°C overnight, embedded in paraffin, and sectioned at 5 μm. Bouin's-fixed sections were deparaffinized, rehydrated, and stained with H&E. For immunofluorescence studies, PFA-fixed sections were boiled in 10 mM sodium citrate buffer (pH 6.0) for 15 min in a microwave oven, brought to RT, washed in phosphate-buffered saline (PBS) with 0.1% Triton X-100 (PBST), and then incubated for 60 min at RT with blocking buffer (10% donkey serum, 1% BSA, and 0.1% Triton X-100 in PBS). The sections were then incubated with the primary antibodies in blocking buffer overnight at 4°C. After washing in PBST, the slides were incubated with a 1:500 dilution of Alexa Fluror 488- or Alexa Fluror 594-conjugated donkey secondary antibody (Jackson ImmunoResearch Laboratories) for 60 min at RT. The sections were washed in PBST, rinsed quickly in 100% ethanol, mounted in Prolong Gold Antifade medium with DAPI (Molecular Probes), and imaged using a fluorescence microscope (Olympus). Rabbit anti-DHX36 (1:100; Abcam), mouse anti-γH2AX (1:200; Millipore), anti-BG4 (1:50; Sigma), and FITC-conjugated peanut agglutinin (1:500; Sigma) were used in this study. TUNEL assay was performed using an In Situ Cell Death Detection kit, Fluorescein (Roche Applied Science) according to the manufacturer's instructions.

### Meiotic chromosome spreading and immunofluorescence staining

Testes were collected from adult wild-type (WT) and *Dhx36-*mutant male mice. Spreads of male germ cell chromosomes were performed as reported previously (Peters et al., 1997). In brief, seminiferous tubules were treated with hypotonic buffer (30 mM Tris, 5 mM EDTA, 50 mM sucrose, 17 mM trisodium citrate dihydrate, and 0.5 mM dithiothreitol, pH 8.2) for a maximum of 30 min and then smashed in 100 mM sucrose buffer (pH 8.2). The suspension was then gently spread onto slides containing fixative buffer (1% PFA, 0.15% Triton X-100, pH 9.2). After 2 h incubation in a humidity chamber at RT, the slides were air-dried and washed in PBS three times before immunofluorescence staining. To stain, the slides were blocked with 10% donkey serum and incubated with primary antibody for 1 h at RT. Slides were then washed and incubated with Alexa Fluor 488- or Alexa Fluor 594-conjugated secondary antibody (Jackson ImmunoResearch Laboratories), mounted, and imaged using a Zeiss LSM710 confocal microscope. Semiquantitative analysis of the fluorescence signals was conducted with the NIH Image program ImageJ.

### Isolation of mouse zygotene spermatocytes

Zygotene spermatocytes were isolated from WT and *Dhx36*-mutant spermatogenesis-synchronous mice using an established approach as described previously (Chen et al., 2018b). Briefly, 2-day-old animals were pipette-fed with WIN18466 (100 μg/g body weight) suspended in 1% gum tragacanth for seven consecutive days. WIN18466-treated mice were then administered via an i.p. injection of all-trans-RA (33 μg/g body weight; Sigma) in dimethyl sulfoxide (Sigma), left to recover for 216 h, and their testes were collected in PBS on ice. After removal of the tunica albuginea, the testes were incubated in 5 ml PBS with 120 U/ml of collagenase type I (Worthington) at 32°C with gentle agitation for 5 min. The dispersed seminiferous tubules were then digested with 5 ml 0.25% trypsin (Gibco) and 0.1 ml DNase I (5 mg/ml; Sigma) at 32°C for 8 min, and the digestion was terminated by adding 0.5 ml of fetal bovine serum (Gibco) to inactivate trypsin. The resulting dissociated testicular cells were filtered through a PBS-prewetted cellular filter (70 μm). After spun down at 500 *g* for 5 min at 4°C, the cells in the pellet were resuspended at a concentration of 1 × 10^6^ cells/ml in DMEM with Hoechst 33342 (3 mg/ml; Sigma) and 5 μl DNase I followed by rotating for 20 min at 32°C in the oven at 10 rpm speed. Zygotene spermatocytes were collected based on their fluorescent label with Hoechst 33342 staining using FACS (BD Biosciences). Cell purity was further examined by meiotic nuclear spreading with immunofluorescence staining as described earlier. The purity of isolated zygotene is ∼95%.

### RNA-seq and qRT–PCR assays

Total RNA was extracted from zygotene spermatocytes isolated from WT and *Dhx36*-mutant spermatogenesis-synchronous mice using Trizol reagent (Invitrogen). RNA quality was determined by Qubit Fluorometer and Agilent Bioanalyzer 2100. rRNAs were removed from total RNAs by the NEBNext rRNA Depletion Kit (NEB). The remaining RNAs were fragmented and then reverse-transcribed. Libraries were prepared by the Omics Core of CAS–MPG Partner Institute for Computational Biology at Shanghai Institute of Nutrition and Health, Chinese Academy of Sciences using NEBNext Ultra II Directional RNA Library Prep Kit for Illumina (NEB) according to the manufacturer's instructions. Libraries were sequenced using single reads on Illumina HiSeq 2000 (Illumina). Quality control (QC) of the reads from high-throughput sequencing pipelines was carried out by FastQC (version 0.11.9). Low-quality bases and adapter-containing reads were then trimmed from raw data by Trim Galore (version 0.6.7) with default parameters. The remaining trimmed sequences were further mapped against the ENSEMBL mouse reference genome (mm9) with HISAT2 (version 2.2.1), which allowed mapping across splicing junctions by read segmentation. The subsequent mapped reads were quantified by FeatureCounts (available in Subread software package, version 2.0.3). All programs were performed with default settings unless otherwise specified. DEGs were identified as those with adjusted *P*-value <0.05 and a fold change >1.5 using Limma-voom (version 3.50.0). GO, KEGG, and GSEA analyses were performed by ClusterProfiler (version 4.2.1) with an adjusted *P*-value <0.05. The reference databases (C2: curated gene sets; C5: ontology gene sets) of GSEA were retrieved from MSigDB.

qRT–PCR was performed using a SuperScript III Platinum SYBR Green One-Step qRT–PCR kit (Invitrogen). Relative expression of genes was analyzed by the comparative C_T_ method with use of ribosomal protein S2 (*Rps2*) as a normalized control. qRT–PCR primer sequences are listed in Supplementary Table S2.

### G4 prediction using pqsfinder

pqsfinder, an R package, can detect DNA and RNA sequence patterns that are likely to fold into an intramolecular G4 (Hon et al., 2017). The prediction of PQS was performed with this package according to its user guide (pqsfinder: User Guide (nju.edu.cn)). The analysis revealed numerous potential G4-forming sites in the selected gene sequences. After examining the potential of such G-runs to form a stable G4, it assigned a corresponding quantitative score to each. All programs were performed with default settings unless otherwise specified. In order to make diagrams more aesthetic and readable, we used Gviz package to optimize them with the manufacturer's guideline (The Gviz User Guide (bioconductor.org)).

### Data access

All the data obtained from RNA-seq have been deposited in Gene Expression Omnibus (GEO) with GSE218477.

### Statistical analyses

For all analyses, data were statistically processed using a Student's *t*-test for all pairs computed by SigmaStat 3.0 (SPSS). A *P*-value <0.05 was considered significant. Data were presented as mean ± standard deviation (SD).

## Supplementary Material

mjac069_Supplemental_Files

## Notes

**Author contributions:** K.Z. carried out most experiments and data analysis; X.T., Yujie Zhang, and J.Y. performed several nuclear spreading experiments; C.Z. and Q.Y. provided *Dhx36* floxed mouse lines; M.-H.T., K.Z., and T.Z. wrote the manuscript. All the authors were involved in the discussion on the manuscript.
