# Supplementary material for: DNA/RNA helicase DHX36 is required for late stages of spermatogenesis
Source: J Mol Cell Biol. 2022 Dec 9;14(11):mjac069. doi: 10.1093/jmcb/mjac069 (PMC10166722; doi:10.1093/jmcb/mjac069)
Supplement: mjac069_Supplemental_Files [file mjac069_supplemental_files.zip › JMCB-2022-0263.R3_Supplementary material.pdf]

Supplementary material

DNA/RNA helicase DHX36 is required for late stages of spermatogenesis

Kejia Zhang[2], Tianxin Zhang[2], Yujie Zhang[2], Jinyu Yuan[2], Xinzhe Tang[2], Chaobao Zhang[2], Qianqian Yin[2], Yonglian Zhang[2], and Ming-Han Tong[1,2,*]

[1] School of Life Science, Hangzhou Institute for Advanced Study, University of Chinese Academy of Sciences, Chinese Academy of Sciences, Hangzhou 310024, China

[2] State Key Laboratory of Molecular Biology, Shanghai Key Laboratory of Molecular Andrology, Shanghai Institute of Biochemistry and Cell Biology, Center for Excellence in Molecular Cell Science, University of Chinese Academy of Sciences, Chinese Academy of Sciences, Shanghai 200031, China

* Correspondence to: Ming-Han Tong, E-mail: minghan@sibcb.ac.cn

Supplementary Figures

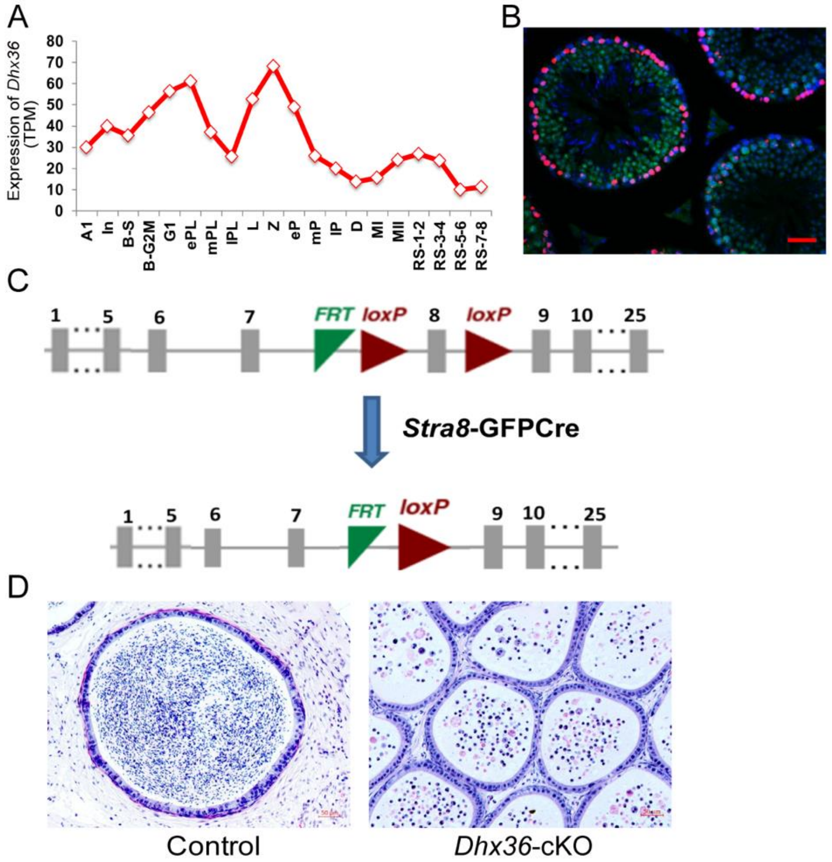

Supplementary Figure S1 (**A**) *Dhx36* transcript levels are shown as transcripts per million (TPM) in different developmental stages of male germ cells. A1: type A1 spermatogonia; In: type In spermatogonia; B-S: S phase

of type B spermatogonia; B: G2/M: G2/M phase of type B spermatogonia; ePL: early preleptotene spermatocytes; mPL: middle preleptotene spermatocytes; lPL: late preleptotene spermatocytes; L: leptotene spermatocytes; Z: zygotene spermatocytes; mP: middle pachytene spermatocytes; lP: late pachytene spermatocytes; D: diplotene spermatocytes; MI: metaphase I; MII: metaphase II; RS-1-2: step 1-2 spermatids; RS-3-4: step 3-4spermatids; RS-5-6: step 5-6 spermatids; RS-7-8: step 7-8 spermatids. (**B**) Immunofluorescence staining for DHX36 (green), DAPI (blue) and γH2AX (red) in sections of adult mouse testis. Scale bar: 40 µm. (**C**) Schematic diagram of *Dhx36* conditional KO allele. (**D**) Morphology of epididymis from control and *Dhx36*-sKO mutant mice. Scale bar: 40 µm.

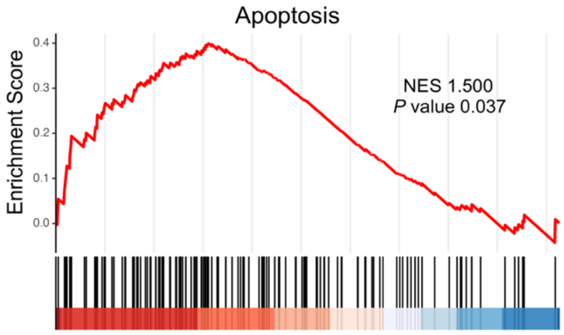

Supplementary Figure S2 Gene set enrichment analyses of all differential expressed genes showing enrichment with apoptosis pathway in *Dhx36*-cKO mutant spermatocytes.

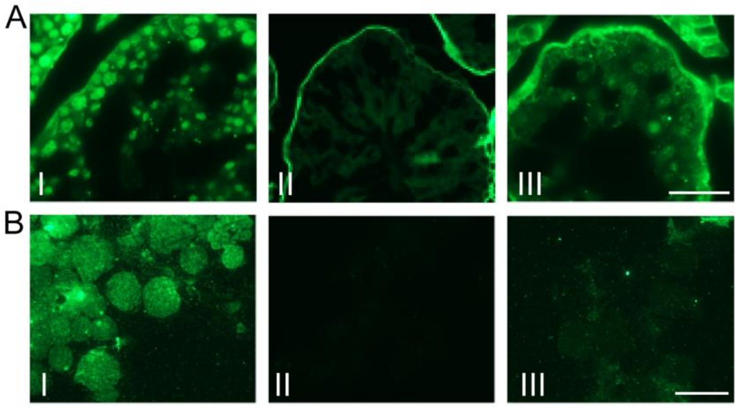

Supplementary Figure S3 (**A**) Immunofluorescence staining for BG4 (green) in sections of adult mouse testis. I: in the presence of the BG4 primary antibody without DNase I; II: in the absence of the BG4 primary antibody; III: in the presence of the BG4 primary antibody with DNase I. Scale bar: 20 µm. (**B**) Immunofluorescence staining for BG4 (green) in isolated spermatocytes from adult mouse testes. I: in the presence of the BG4 primary antibody without DNase I; II: in the absence of the BG4 primary antibody; III: in the presence of the BG4 primary antibody with DNase I. Scale bar: 10 µm.

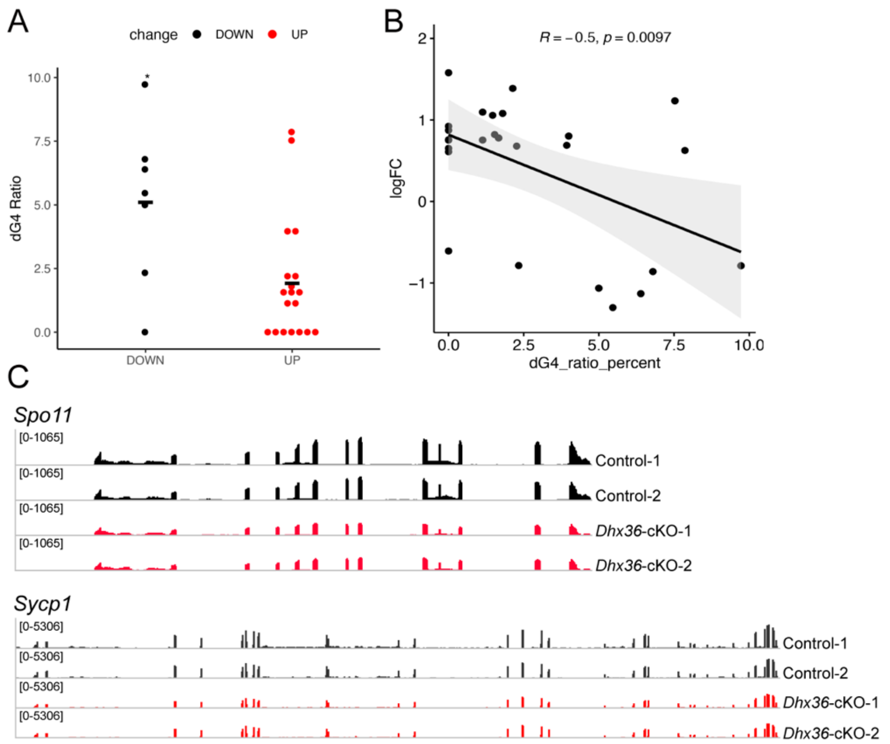

Supplementary Figure S4 (**A**) Dotplot showing the difference of G4 enrichment in the promoter regions between downregulated meiosis-associated genes and upregulated meiosis-associated genes in *Dhx36* deficient spermatocytes. dG4 ratio defined as the percentage of putative PQS in 1.5kb ahead of transcriptional start site (TSS) in genes' template strand. (**B**) Correlation analysis between dG4 ratio of meiosis-associated DEGs and their fold changes on mRNA levels in the zygotene spermatocytes upon *Dhx36* deficiency. (**C**) RNA read density distributions along the coding sequence of *Spo11* and *Sycp1* as an example in zygotene spermatocytes.

Supplementary Tables

Supplementary Table S1 A list of 3555 DEGs (1826 up and 1729 down) exhibited significant changes (P-value <0.05, >1.5-fold difference) in mutant zygotene spermatocytes compared to that in controls.

Supplementary Table S2 A list of qRT–PCR primer sequences.
